# Tracing the Rise of Galectin-3: A Global Bibliometric Insight Into Its Role in Heart Failure

**DOI:** 10.1155/ijvm/7431078

**Published:** 2025-07-24

**Authors:** Amjad Bani Hani, Alaa Tarazi, Lubna Alnatour, Ahmad Aburrub, Mohammad Hijah

**Affiliations:** ^1^General Surgery Department, School of Medicine, The University of Jordan, Amman, Jordan; ^2^School of Medicine, The University of Jordan, Amman, Jordan

**Keywords:** atherosclerosis, bibliometric, biomarker, galectin-3, heart failure

## Abstract

**Background:** Heart failure (HF) is a growing clinical syndrome with high morbidity and mortality. Galectin-3, a key player in cardiac fibrosis and inflammation, has emerged as an important biomarker for HF. This bibliometric analysis is aimed at exploring global scientific output and research trends on the relationship between galectin-3 and HF.

**Methods:** A bibliometric literature search was conducted on the Web of Science in September 2024. Microsoft Excel and VOSviewer were used for scientometric analysis and to visualize scientific achievements, including publication counts, key authors, countries, organizations, journals, and research hotspots in the field.

**Results:** A total of 705 publications met the inclusion criteria after screening. Research on galectin-3 and HF is currently experiencing rapid growth. The United States, China, and the Netherlands produced the most articles, contributing approximately 55% (388/705) of all papers. Most institutions and authors were from the United States and the Netherlands, with the University of Groningen (Netherlands) being the top publishing institution. Key authors include De Boer RA, Januzzi JI, and Van Veldhuisen DJ. The *European Journal of Heart Failure* was the most cited journal and had the highest number of publications. Key research topics include the relationship between galectin-3 and HF prognosis, fibrosis, mortality, and conditions leading to HF.

**Conclusion:** This is the first bibliometric analysis of publications on the association between galectin-3 and HF. This study provides researchers with valuable insights into the most influential articles on this biomarker and its role in HF. Key research areas focus on galectin-3 role in the diagnosis, pathology, and prognosis of various HF types, causes, and outcomes. Further research should explore how galectin-3 can facilitate earlier diagnosis of HF or fibrosis, with increased international collaboration among researchers.

## 1. Introduction

Heart failure (HF) is a leading cause of mortality, morbidity, and reduced quality of life [1]. It is an escalating public health concern, affecting approximately 40 million people worldwide and representing a major cause of hospital admissions among middle-aged and elderly populations [2]. HF is a chronic, progressive condition characterized by structural or functional abnormalities of the heart, resulting in either reduced (heart failure with reduced ejection fraction, HFrEF) or preserved (heart failure with preserved ejection fraction, HFpEF) left ventricular ejection fraction (LVEF) [3].

Diagnosing HF is challenging due to its nonspecific symptoms, which often overlap with those of other medical conditions [4]. Circulating biomarkers reflecting key pathophysiological pathways in HF development and progression can aid in early diagnosis and treatment [5]. Among these, galectin-3 (gal-3) has emerged as a crucial biomarker due to its role in cardiac fibrosis, inflammation, and ventricular remodeling [6]. Recent studies have linked gal-3 to HF progression, demonstrating its association with disease severity [7].

This study is aimed at providing a comprehensive, up-to-date analysis of gal-3, a novel biomarker in HF, using bibliometric analysis. By examining current research trends and developments, this study seeks to predict the future research landscape of gal-3. Additionally, it will highlight key areas of interest, helping researchers navigate the field more efficiently. Ultimately, a deeper understanding of the relationship between gal-3 and HF may contribute to improved strategies for preventing HF complications.

## 2. Methods

### 2.1. Data Collection

Data were collected from the Web of Science Core Collection (WoSCC) database on September 13, 2024, using the search parameters “full record and cited references” and “plain text.” WoSCC was selected for its extensive repository of high-quality, peer-reviewed literature from around the world [8]. The search strategy employed the formula: TS = (“Galectin-3” OR “Gal-3”) AND TS = (“heart failure” OR “HF”). Only articles and reviews were included. To ensure the accurate selection of studies investigating the role of gal-3 in HF, all retrieved literature was screened based on titles and abstracts. Any discrepancies were resolved through thorough discussion until consensus was reached.

**Figure 1:**
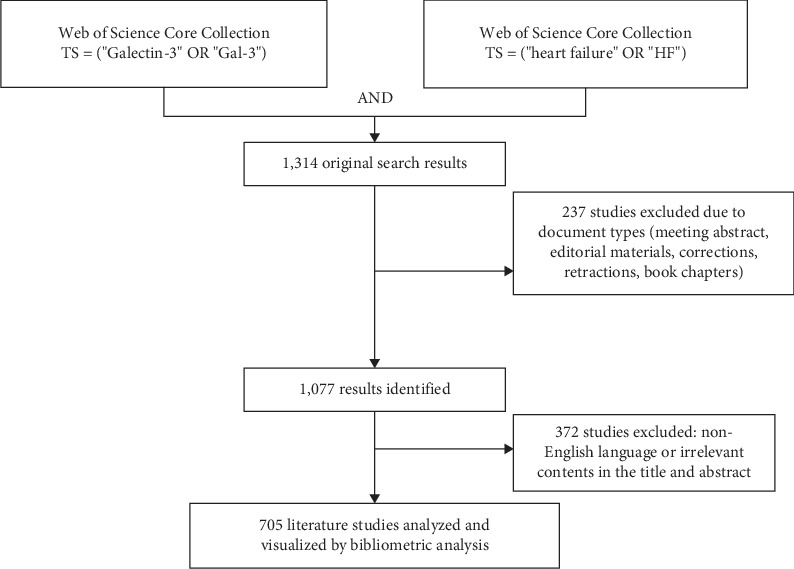
Screening processing flowchart.

The initial search yielded 1314 articles. After excluding entries outside the articles and reviews category, 1077 articles remained. Further screening excluded non-English articles and those unrelated to our focus on the role of gal-3 in HF, resulting in 705 articles for analysis. A detailed overview of the screening process is presented in Figure 1.

### 2.2. Data Analysis

Microsoft Excel 2019 and VOSviewer (Centre for Science and Technology Studies, Leiden University, The Netherlands) were used to analyze 705 publications. Bibliometric data, including titles, keywords, authors, countries, institutions, journals, citations, and publication years, were systematically extracted from the WoSCC database.

VOSviewer (Version 1.6.20) was used to identify key contributors, including productive authors, countries, and institutions, as well as to perform keyword co-occurrence analysis. This software is widely recognized for generating bibliometric visualization maps, where frequently occurring terms are represented by larger bubbles, and terms with high similarity are placed in close proximity [9]. The extracted data were saved in .txt format and imported into VOSviewer for analysis. Various analyses—including coauthorship, co-occurrence, and citation—were conducted by selecting the appropriate unit of analysis (e.g., authors, countries, institutions, keywords, or sources). The processed data were then visualized and analyzed.

Microsoft Excel was used to manage and organize data, create tables, and generate trend charts to visualize annual publication trends. It also facilitated data screening after extraction from the WoSCC database. Additionally, results from VOSviewer were exported to Excel for further organization, enabling the creation of comprehensive final tables.

## 3. Results

### 3.1. Documents per Year

**Figure 2:**
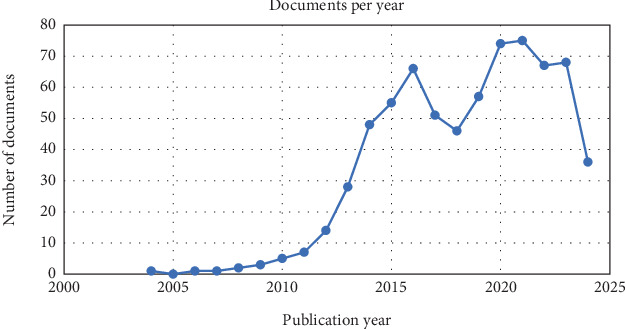
Number of documents among years.

Over the past two decades, the number of publications examining the biomarker gal-3 in relation to HF has steadily increased. The first study was published in 2004, and the volume of research grew consistently until 2018, when a notable decline occurred compared to previous years. However, after 2018, the number of publications gradually rose again, reaching a peak in 2021 with 75 publications, as illustrated in Figure 2.

### 3.2. Authors

A total of 4471 authors have actively contributed to the research field of HF and gal-3 as outlined in Table 1. Rudolf de Boer stands out as the most prolific author with 44 publications representing 6.24% of the overall literature. Moreover, he was reported as the most cited author. He is affiliated with the Thoraxcenter, Erasmus MC, in the Netherlands. Close on his heels is James Januzzi, with 27 publications to his name, who is affiliated with Harvard Medical School and the Baim Institute for Clinical Research in Boston, Massachusetts, United States. Following closely is Dirk J. van Veldhuisen with 26 publications. Interestingly, the United States led the list of countries with the highest number of publications, although most of the top 10 authors were affiliated with Dutch institutions.

**Figure 3:**
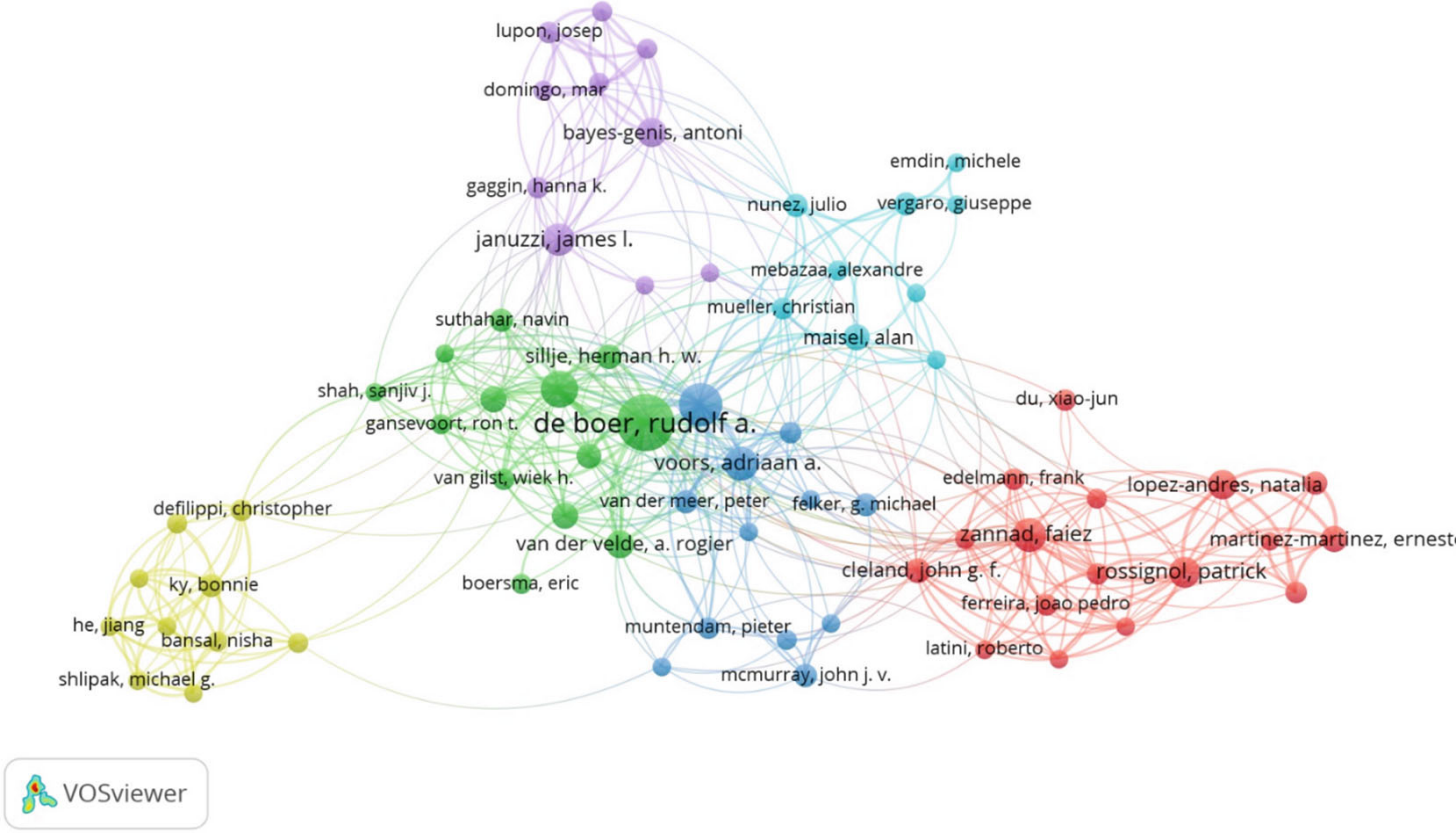
Visualization map of the most productive authors in the publication of gal-3 in association with HF.

To visualize this network of authors, a map generated using VOSviewer (1.6.20) where authors are represented as nodes, with larger nodes denoting those with a higher publication count is presented in Figure 3. This map illustrates the collaborative network among authors in the HF field. For instance, Rudolf de Boer, RA de Boer, having the largest node has engaged in collaborations with numerous authors such as Antoni Bayes-Genis and Eric Boersma. Also, thicker lines connecting to other nodes indicate the extent of authors' collaborations and the strength of these connections.

### 3.3. Institutions

Table 2 presents a list of the top 10 most prolific institutions in the field of gal-3 as a biomarker for HF. Together, these institutions account for 29.93% of the articles published in this area. The leading institute in terms of publications is the University of Groningen (Netherlands) with 62 publications (8.80%), followed by the Massachusetts General Hospital (United States) with 26 publications (3.69%). The University of California San Diego (United States) ranked as the third institute with 22 publications (3.12%).

Notably, all of the top 10 institutions were located in four regions: Netherlands, the United States, the United Kingdom, and France. Collectively, American institutions ranked as the most productive, contributing a total of 124 documents among the top 10 organizations, followed by the Netherlands, which accounts for 62 documents in the top 10 institutions.

**Figure 4:**
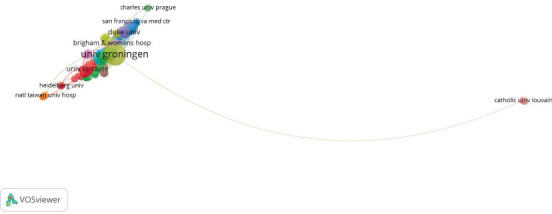
Visualization map of the most productive institutions in the publication of gal-3 in association with HF.

Collaborations are more common among institutions that are geographically close to each other as shown in Figure 4. This can be illustrated by the collaboration between the Brigham and Women's Hospital, the Duke University, and the San Francisco VA Medical Center.

### 3.4. Countries

**Figure 5:**
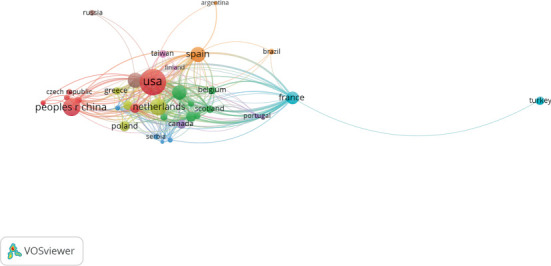
Visualization map of the most productive countries in the publication of gal-3 in association with HF.

Researchers from 72 countries were involved in the studies in this field. The United States ranked as the most significant contributor in this field with 204 documents, accounting for (28.96%) of the overall literature and amassing 10,290 total citations. China ranked second with 96 publications and 2311 total citations, followed by the Netherlands with 88 publications and 17,7131 total citations, as illustrated in Table 3. This suggests that gal-3 in HF has played a critical role in cardiovascular research in these three countries. VOSviewer was used to visualize the collaborative network among these countries, with each node representing a specific country and lines depicting the collaborations between them, as shown in Figure 5. Numerous collaborations among different countries, such as the United States, Spain, Netherlands, China, and Scotland, are evident.

### 3.5. Journals

**Figure 6:**
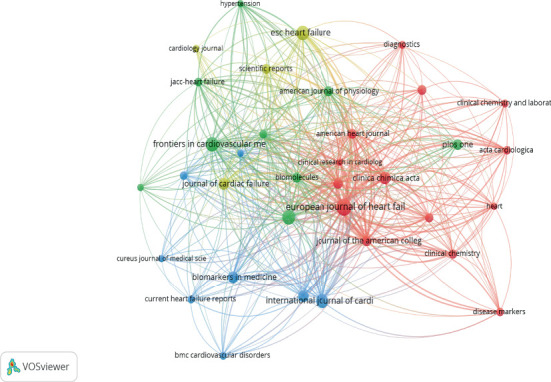
Network visualization map of the top productive journal in the publication of gal-3 in association with HF.

The top 10 journals have collectively published 170 articles, representing 24.1% of the total studies included in this review, and have garnered 6788 citations. The *European Journal of HF* ranks first both in terms of publication volume and citation impact, with 27 articles and 2460 citations. It is followed by the *International Journal of Cardiology* and the *Journal of the American Heart Association*, each contributing 20 articles, as shown in Table 4. Among the top 10 journals, most are classified in Quartiles 1 and 2 according to the Journal Citation Report (JCR) 2024, with the exception of *Biomarkers in Medicine*, which falls into Quartile 3. A network visualization map illustrating the active journals in the field of gal-3 in association with HF is presented in Figure 6.

### 3.6. Keywords

Keyword co-occurrence analysis is a valuable tool for identifying patterns in research topics, trends, and the conceptual structure of a specific field.  Table 5 presents the top 20 keywords related to gal-3 in association with HF. The most frequently occurring keyword is “Heart Failure,” appearing 476 times, followed by “Galectin-3” with 380 occurrences and “Biomarker” with 320 occurrences. These findings highlight the centrality of these terms and their prominence in the included studies.

**Figure 7:**
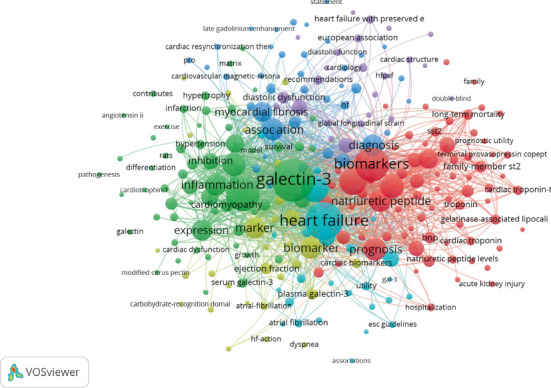
Network map of the keyword clusters in the studies of gal-3 in association with HF.

Keyword clusters are groups of related keywords that offer insights into the structure and trends of research within a field, helping to uncover key themes, concepts, and emerging areas of interest. Each cluster represents a distinct research frontier. In this study, six clusters were identified, as shown in Figure 7

## 4. Discussion

### 4.1. General Data

Over the past two decades, there has been a steady global increase in the recognition of gal-3 in relation to HF, following its initial association with the condition in 2004. This trend may be attributed to the growing evidence that gal-3 is linked to key mechanisms in HF, including cardiovascular fibrosis, inflammation, and its role in diagnostic detection of the disease [6, 10].

### 4.2. Authors

Leading authors include Rudolf de Boer, RA de Boer, from the Thoraxcenter (Netherlands), James Januzzi from both the Baim Institute for Clinical Research and Harvard Medical School (United States), and Dirk J. van Veldhuisen from the University of Groningen (Netherlands). By focusing on these authors, other researchers in the field can gain valuable insight into the current hotspots and emerging trends in the field.

Rudolf de Boer focused on studying the role of gal-3 in predicting the prognosis of HF as it correlates with the degree of cardiac remodeling [11].

James Januzzi's research in proteomics was aimed at refining HF management, with a particular focus on gal-3 [12]. The two authors collaborated on a project to study the correlation between elevated plasma gal-3 and near-term rehospitalization in HF patients [13]. Dirk J. van Veldhuisen contributions center on exploring the correlation between cardiac magnetic resonance imaging (CMRI) findings and gal-3 to predict the outcome in HF [14].

### 4.3. Institution and Countries

The study's findings revealed the United States, China, and Netherlands as the most productive countries concerning publications on gal-3 in HF. The top three contributing institutions in terms of publications hailed from the United States and the Netherlands. Although the United States claimed the top position as the most productive country, it was outperformed by the top-performing institution, the University of Groningen, from the Netherlands, with a total of 62 (8.80%) publications. However, the top three institutions in the United States, which are Massachusetts General Hospital, University of California San Diego, and Duke University, collectively contributed to 65 (9.22%) publications.

Establishing long-term collaborative relationships across international borders is crucial for fostering further advancement in this area.

A highly cited paper exploring gal-3 as a novel mediator in HF development and progression resulted from a collaboration between researchers from the United States and the Netherlands, emphasizing the strong research ties between these two countries. Notably, all Dutch authors in this study were affiliated with the University of Groningen, which ranked first among the top 10 institutions in this field [15]. Moreover, a highly influential paper that focused on HF with reduced ejection fraction and its association with gal-3 resulted from a collaboration among authors from multiple countries, including Germany, Austria, and the United States. Although Germany ranked fifth among the top 10 most cited countries, no German institutions were included in the top 10 institutions [16].

Spain has demonstrated a particular interest in the role of inhibiting gal-3 and the resulting effects on cardiac inflammation and fibrosis. Notably, Spanish researchers have explored how gal-3 inhibition mitigates pathological processes in experimental models of hyperaldosteronism and hypertension. Despite not ranking among the top 10 institutions, Spain's contributions highlight its commitment to understanding the therapeutic potential of targeting gal-3 in cardiovascular disease [17].

France, on the other hand, has focused on understanding the role of gal-3 in promoting vascular smooth muscle fibrosis, a process that can ultimately contribute to HF. Notably, French researchers collaborated on this project with Rudolf A. de Boer, a leading Dutch expert in the field [18]. This partnership underscores the strong international collaboration between top countries and institutions in advancing research on gal-3 and its cardiovascular implications [18].

Chinese researchers have shown particular interest in the prognostic value of plasma gal-3 levels in patients with chronic HF and coronary heart disease. Their studies concluded that gal-3 is an independent predictor of all-cause mortality and rehospitalization, emphasizing its potential role in guiding risk stratification and clinical management of these patients [19].

### 4.4. Journals

Most of the journals in the top 10 rankings are classified as Q1 or Q2, indicating that the majority of articles published on gal-3 in association with HF are of high quality, offering valuable insights and well-supported conclusions. Many of these journals focus specifically on HF within the cardiovascular field, highlighting the expertise of the scholars and the thoroughness of the research. The *European Journal of Heart Failure*, the most cited and prolific journal in this area, has contributed numerous highly relevant articles on this topic [15, 20, 21]. These findings provide cardiologists with valuable guidance on where to find and publish high-impact, relevant research related to biomarkers in HF, ensuring both efficacy and relevance in their work.

### 4.5. Keywords and Hotspots

Keyword visualization and distribution provide valuable insights for authors, helping them identify the key frontiers and emerging trends in a specific research area. In this case, the current research hotspots and trends related to gal-3 in association with HF were identified through the analysis of 130 keywords, each occurring more than 10 times. These keywords were grouped into six distinct clusters using VOSviewer (Version 1.6.20)

#### 4.5.1. Cluster #1, Biomarkers in Acute HF: Diagnostic and Prognostic Insights

The main keywords in this cluster include heart failure, acute heart failure, acute dyspnea, biomarkers, brain natriuretic peptide (BNP), soluble ST2, troponin, and gal-3. These keywords are closely related due to their roles in the diagnosis, prognosis, and management of HF, particularly in acute settings. BNP is primarily used for diagnostic purposes in HF, especially to differentiate HF as the cause of dyspnea from other potential causes [22]. Soluble ST2 serves as a biomarker for cardiac inflammation and has predictive value for the progression of chronic or worsening HF [23]. Additionally, troponin is useful for identifying acute myocardial stress, which can exacerbate or complicate HF [24].

#### 4.5.2. Cluster #2, Advances in Cardiovascular Disease Diagnosis: The Role of MRI and Clinical Guidelines

The main topics of this cluster include cardiovascular diseases, diagnosis, clinical guidelines, recommendations, magnetic resonance imaging (MRI), cardiac magnetic resonance (CMR), and dilated cardiomyopathy. These elements are interconnected through the pivotal role of imaging techniques, such as MRI, in diagnosing cardiovascular diseases. This diagnostic approach is often reflected in clinical guidelines and recommendations. For instance, a study conducted to support the inclusion of CMR in the European Society of Cardiology (ESC) guidelines highlights its utility in diagnosing a range of cardiovascular conditions, including inflammation, cardiomyopathies, and ischemic heart disease [25].

#### 4.5.3. Cluster #3, gal-3 as a Biomarker and Therapeutic Target in HF and Cardiovascular Diseases

The primary keywords in this cluster include galectin-3, heart failure, cardiac fibrosis, inflammation, and myocardial infarction. gal-3 connects HF, cardiac fibrosis, inflammation, and myocardial infarction through its role in the inflammatory response and tissue remodeling processes. Elevated levels of gal-3 are associated with poor prognosis in HF [6, 26]. Additionally, gal-3 has emerged as a potential target for therapeutic strategies aimed at reducing fibrosis and improving outcomes in cardiovascular diseases [27].

#### 4.5.4. Cluster #4, gal-3 as a Prognostic Biomarker in HF and Cardiovascular Disease

The main topics in this cluster include galectin-3 levels, mortality, fibrosis, marker, and ventricular ejection fraction. These keywords highlight the role of gal-3 as a biomarker in cardiovascular disease and its connection to heart function and prognosis. Elevated gal-3 levels are associated with reduced ventricular ejection fraction and increased mortality in HF and other cardiovascular conditions [28, 29]. Furthermore, gal-3 has been shown to provide valuable prognostic information regarding disease progression and risk [10]. It can help identify patients at higher risk of readmission or death from HF and may guide decisions about the frequency of patient monitoring [30, 31]. This underscores the importance of gal-3 in both the diagnosis and prognosis of cardiovascular diseases, as reflected in the articles included in this cluster.

#### 4.5.5. Cluster #5, Plasma gal-3 as a Biomarker in Atrial Fibrillation and HF

The primary topics in this cluster include atrial fibrillation, biomarker, cardiovascular disease, chronic heart failure, congestive heart failure, plasma galectin-3, and ventricular dysfunction. This group of keywords highlights the role of plasma gal-3 as a key biomarker in cardiovascular diseases, particularly in conditions like atrial fibrillation, chronic HF, and congestive HF. Elevated levels of gal-3 have been shown to reflect ongoing fibrosis and inflammation, which contribute to ventricular dysfunction and poor prognosis in these conditions [32, 33]. This underscores the large body of research supporting the efficacy of gal-3 as a biomarker for various cardiovascular diseases.

#### 4.5.6. Cluster #6, gal-3 as a Biomarker in Atherosclerosis, Cardiorenal Syndrome, and Chronic Kidney Disease

This cluster includes the following keywords: atherosclerosis risk, cardiorenal syndrome, chronic kidney disease, and galectin-3. A study conducted by Blanda et al. reported that elevated levels of gal-3 are associated with atherosclerosis and play a key role in the pathogenesis of cardiovascular diseases [34]. Additionally, higher circulating levels of gal-3 have been linked to an increased risk of developing chronic kidney disease and to a more rapid decline in renal function over time [35, 36]. Furthermore, a cohort study done by Ghorbani et al. found that longitudinal changes in gal-3 are associated with cardiovascular and renal risk factors and independently predict future HF, cardiovascular disease, and mortality in the community [37]. These findings highlight the significance of gal-3 not only in cardiovascular diseases but also in renal health, suggesting that future research could explore its role more broadly across both systems.

## 5. Conclusion

This study offers the first comprehensive analysis of gal-3 as a specific biomarker in HF, examining it from both a visualization and bibliometric perspective. Rudolf de Boer emerged as the most prolific author in this field. Additionally, the University of Groningen and the United States stand out as the leading institutions and countries, respectively. The *European Journal of Heart Failure* was identified as the most prominent journal in the area. Research on cardiac fibrosis and cardiac remodeling has increasingly focused on gal-3 as a key biomarker, aiming to reduce their incidence through early detection and intervention. However, further studies are required to deepen our understanding and enhance clinical applications.

## 6. Limitation

The study has several limitations. First, only literature indexed in the WoSCC was included, and it focused solely on two types of publications: articles and reviews. Additionally, the majority of the literature was in English, which may introduce some bias in the selection. While two researchers conducted the screening process, personal opinions, knowledge, and backgrounds could still influence the results. Despite these limitations, this remains the first bibliometric study to assess the role of this specific biomarker in the context of HF.

## Figures and Tables

**Table 1:** The top 10 most productive authors in articles correlated with HF and gal-3.

**Rank**	**Author**	**Institute (country)**	**Documents**	**Total citations**
1	Rudolf de Boer	Thorax Center, Erasmus MC, Rotterdam, Netherlands	44 (6.24%)	4142
2	James Januzzi	Harvard Medical School, Boston, Massachusetts, United States, and Baim Institute for Clinical Research, Boston, Massachusetts, United States	27 (3.83%)	1044
3	Dirk J. van Veldhuisen	University of Groningen, Netherlands	26 (3.69%)	2771
4	Wouter C. Meijers	University of Groningen, Netherlands, and Erasmus Medical Center, Netherlands	20 (2.84%)	1011
5	Adriaan Voors	University of Groningen, Netherlands	16 (2.27%)	1553
6	Faiez Zannad	Université de Lorraine, Inserm, Centre d'Investigation Clinique Plurithématique, France	16 (2.27%)	1299
7	Patrick Rossignol	Université de Lorraine, Inserm, Centre d'Investigation Clinique Plurithématique, France	14 (1.99%)	796
8	Antoni Bayes-Genis	Hospital Germans Trias i Pujol, Badalona, Spain	12 (1.70%)	577
9	Natalia Lopez-Andres	Instituto de Investigación Sanitaria de Navarra (IdiSNA), Spain	12 (1.70%)	994
10	A. Rogier Van Der Velde	University of Groningen, Netherlands	11 (1.60%)	616

**Table 2:** Top 10 most prolific institutions about gal-3 in association with HF.

**Rank**	**Institute**	**Country**	**Publications (** **N**/705**)**
1	University of Groningen	Netherlands	62 (8.80%)
2	Massachusetts General Hospital	United States	26 (3.69%)
3	University of California San Diego	United States	22 (3.12%)
4	Duke University	United States	17 (2.41%)
5	University of California, San Francisco	United States	17 (2.41%)
6	University of Lorraine	France	17 (2.41%)
7	University of Maryland	United States	16 (2.27%)
8	University of Glasgow	United Kingdom	15 (2.13%)
9	Harvard Medical School	United States	14 (1.99%)
10	Brigham and Women's Hospital	United States	12 (1.70%)

**Table 3:** Top 10 most productive countries about gal-3 in association with HF.

**Rank**	**Country**	**Publications (** **N**/705**)**	**Citations**
1	United States	204 (28.96%)	10,290
2	China	96 (13.69%)	2311
3	Netherlands	88 (12.48%)	7131
4	Italy	67 (9.50%)	2412
5	Spain	67 (9.50%)	2285
6	Germany	64 (9.08%)	2987
7	France	50 (7.09%)	2420
8	England	33 (4.68%)	1472
9	Poland	31 (4.40%)	591
10	Australia	28 (3.97%)	785

**Table 4:** Top 10 most productive journals in gal-3 in association with HF.

**Rank**	**Journal**	**Documents**	**Total citations**	**Quartile in category**
1	*European Journal of Heart Failure*	27	2460	Q1
2	*International Journal of Cardiology*	20	618	Q2
3	*Journal of the American Heart Association*	20	620	Q1
4	*ESC Heart Failure*	19	188	Q2
5	*Frontiers in Cardiovascular Medicine*	19	138	Q2
6	*Clinical Chimica Acta*	15	641	Q2
7	*American Journal of Cardiology*	13	519	Q2
8	*Biomarkers in Medicine*	13	175	Q3
9	*Journal of Cardiac Failure*	13	275	Q1
10	*Circulation-Heart Failure*	11	1154	Q1

**Table 5:** The top 20 keywords in articles correlated with gal-3 in association with HF.

**Rank**	**Keyword**	**Frequency**
1	Heart failure	476
2	Galectin-3	380
3	Biomarker	320
4	Fibrosis	216
5	Prognostic value	151
6	Mortality	143
7	Brain natriuretic peptide	136
8	Natriuretic peptide	133
9	Marker	108
10	Prognosis	104
11	Dysfunction	101
12	Association	98
13	Diagnosis	91
14	Inflammation	91
15	Expression	83
16	Soluble ST2	82
17	Myocardial fibrosis	77
18	Disease	67
19	Macrophages	59
20	Risk	57

## Data Availability

The data that support the findings of this study are available from the corresponding author upon reasonable request.

## Nomenclature

gal-3: galectin-3
HF: heart failure
WoSCC: Web of Science Core Collection
BNP: brain natriuretic peptide
MRI: magnetic resonance imaging
JCR: Journal Citation Report
